# Physical–Chemical and Microbiological Characterisation of Blueberry By-Products (*Vaccinium myrtillus* L.) as Potential Food Ingredients

**DOI:** 10.3390/foods15101800

**Published:** 2026-05-19

**Authors:** Miriam Ortega-Heras, Mª Luisa González-Sanjosé, Ruth Hortigüela-Delgado, Ángela Fernández-Varona, Verónica Rodríguez, Beatriz Melero

**Affiliations:** Department of Biotechnology and Food Science, University of Burgos, Plaza Misael Bañuelos, 09001 Burgos, Spain; marglez@ubu.es (M.L.G.-S.); bmelero@ubu.es (B.M.)

**Keywords:** *Vaccinium myrtillus* L., blueberry pomace, anthocyanins, phenolic compounds, product safety, heat treatment

## Abstract

The production of blueberry juice generates large quantities of waste products such as skins, pulp and seeds. These by-products contain high levels of bioactive compounds and are suitable for use as functional ingredients in food systems. In this study three powdered products obtained from blueberry pomace—with skins and seeds, seedless, and with seeds—have been physically–chemically and microbiologically characterised as being the individual anthocyanins and phenolic compounds identified by HPLC-QTOF. Furthermore, to ensure product safety, the effect of a heat treatment at 90 °C for 30, 60, 90 and 120 min was also studied. The three products showed high concentrations of fibre, phosphorus, calcium and potassium. The two products with skins also showed high levels of anthocyanins, phenolic compounds and antioxidant activity. The product from seeds had the highest load of Aerobic Mesophilic Bacteria and *Enterobacteriaceae* whereas *Bacillus* spp. was found in the three products. Heat treatment at 90 °C for 90 min reduced the Aerobic Mesophilic Bacteria count below the detection limit. This treatment did not significantly affect the anthocyanin content, although some anthocyanins seemed to be more thermolabile than others, and increased the concentration of the phenolic acids and their derivatives. This study demonstrates the high nutritional and functional value of blueberry by-products, highlighting their potential as sustainable ingredients in the food industry and their viability after a heat treatment.

## 1. Introduction

Food waste is a significant global challenge. Approximately one-third of all food produced for human consumption is lost or wasted each year [1]. This fact affects many sectors of the food industry, including meat, fresh fruit, vegetables, and processed foods. It has severe environmental, economic and social consequences. Various strategies have been proposed across the food industry to address these problems. One of these strategies involves the revaluation of by-products generated by some of these industries due to their high bioactive compound content. Waste generated by food industries involved in producing juices, liqueurs and jams from berries is of great interest due to their high bioactive compound content. Blueberries (*Vaccinium myrtillus* L.) are among the most widely consumed berries worldwide. They can be consumed fresh or processed into juices, jams, or liqueurs. However, a significant amount of waste is generated during the manufacturing process of these products, mainly in the form of seed and skin, that can account for 20–30% of the processed raw material [2].

This pomace is rich in the following bioactive compounds: fibre, typically exceeding 20% in pomaces. This fibre can bind to phenolic compounds with antioxidant capacity [3]; organic acids (citric and ascorbic acid); sugars (glucose, fructose and galactose); vitamins (A and E); and minerals (K, Ca, Mg, P, Fe, Mn and Zn) [4,5,6]. It is well known that vitamins and minerals are essential for many bodily functions and must be present in the diet. They are also one of the most important sources of phenolic compounds, such as phenolic acids and flavonoids [7,8]. Phenolic acids are mainly derivatives of p-hydroxybenzoic acid and p-hydroxycinnamic acid. More than eleven kinds of phenolic acids have been identified in blueberries, including chlorogenic, gallic, caffeic, cinnamic, ferulic, protocatechuic and p-coumaric acids [5]. Flavonoids are phenolic compounds with a backbone of 15 carbon atoms arranged in a C6-C3-C6 structure. They consist of two aromatic rings linked by a three-carbon heterocyclic ring containing oxygen. The main flavonoids found in blueberry are anthocyanins, flavonols and flavanols. Blueberry is especially rich in anthocyanins and water-soluble flavonoids than can react with glucose, rhamnose, galactose, and arabinose and are capable of forming five kinds of typical anthocyanins, including delphinidin, malvidin, peonidin, cyanidin, and petunidin [9]. The main flavonols identified in bilberries have been quercetin, myricetin, laricitrin, syringetin and isorhamnetin glycosides, whereas the main flavanols found in blueberry are catechin, epicatechin and proanthocyanidis [10]. All these phenolic compounds are powerful antioxidants with health-promoting effects [11]. Flavonoids have demonstrated anti-inflammatory properties that can help mitigate the inflammatory processes in the body. Furthermore, these compounds may inhibit the growth of cancer cells and promote apoptosis, making them a potential component in cancer prevention Flavonoids have also been linked to improving cardiovascular health due to their ability to reduce oxidative stress and inflammation in blood vessels, which can help prevent atherosclerosis and reduce the risk of heart diseases. They have also shown promise in protecting the brain from oxidative damage and inflammation, potentially reducing the risk of neurodegenerative diseases such as Alzheimer’s and Parkinson’s and they may help manage diabetes by improving insulin sensitivity and reducing inflammation [12].

Apart from their nutritional and health benefits, the polyphenolic compounds present in blueberry pomace have various applications in the food industry. For instance, they can be used as colourants thanks to their high anthocyanin content [13] and as preservatives due to their antimicrobial properties [14,15], as well as their ability to delay lipid oxidation [16]. Therefore, they could be used to produce healthier and clean-label foods although the low bioavailability of these compounds presents challenges for their utilisation. However, this may also present opportunities for further research into their biological effects [17]. Furthermore, most studies published on the characterisation of powdered blueberry pomace products focus solely on the whole pomace (skins and seeds). Products obtained from blueberry pomace, whether liquid or powdered, have been used in the food industry to produce dairy products, fermented beverages, pastries, baked goods, snacks, plant-based meat products, and dietary supplements [3,17,18]. However, adding pomace to food formulations can cause sensory changes in terms of colour, flavour and texture. Nevertheless, several foods containing blueberry pomace are accepted by consumers [3]. Finally, these products have also been used in packaging films and intelligent packaging [3,19]. However, these studies focus solely on the whole pomace (skins and seeds).

Another challenge associated with the use of products obtained from blueberry pomace is its high moisture content (around 60%); it is therefore necessary to subject it to a drying process prior to any further treatment, with the aim of improving its physical-chemical, biological and microbiological stability. Hot-air convection drying is the traditional method used to produce powdered products from blueberry pomace and other berries. Most published studies indicate a significant reduction in phenolic compounds, mainly anthocyanins, following such treatments at temperatures above 60 °C [3]. However, ref. [20] demonstrated the effectiveness of convective drying at 70 °C for the drying of blueberry pomace, as the techno-functional properties and bioactive content of the pomace were not significantly altered. Calabuig et al. [21] also found that increasing the drying temperature (from 60 °C to 70 °C) did not significantly affect the polyphenol content or antioxidant activity of blueberry pomace. In recent years, other drying methods have been studied to reduce the moisture content of various berries and their pomace, such as microwave-assisted extraction, electrohydrodynamic drying, freeze-drying, pulsed vacuum drying, heat pump drying, and infrared drying [3,22,23]. However, these methods do not always yield the expected results, and they also involve high installation and operating costs [3]. In this sense, ref. [22] studied the effects of hot-air drying, microwave-assisted hot-air drying and vacuum freeze drying on the antioxidant and volatile profiles of blueberry pomace. They concluded that concerning the cost, antioxidant and aroma profiles, hot-air drying at 60 °C is a suitable technique for the drying of blueberry pomace. However, none of these studies have considered the effect of these drying treatments on the microbiological quality of products obtained from pomace. Considering, that the ultimate purpose of these powdered products is their addition to foodstuffs, they can be considered as spices. Although Regulation (EC) 2073/2005 [24] does not include microbiological hygiene or food safety parameters for spices, it is in the processing industry’s interest to ensure that these by-products are as safe as possible. This would help maintain food safety, protect consumers from foodborne toxins and ensure a low initial microbiological load, thereby avoiding reducing the shelf life of the foods to which they are added. In this sense, although blueberry by-products are dried before milling, reducing their humidity below 10%, the thermal treatments applied are usually below 60 °C [25]. This temperature is insufficient to reduce the microbiological load to an acceptable level when added to foodstuffs; thus, a further thermal treatment at a higher temperature may be required [26]. In the literature, studies characterizing blueberry byproducts focus on their composition in terms of phenolic compounds, fibre, trace elements, and antioxidant activity; however, their microbiological load is not routinely characterized. Goldmeyer et al. [27] found only a low load of moulds and yeast (8.25 ufc/g) in blueberry flour obtained directly from the whole fruit and dehydrated at 60 °C during 36 h.

For all the above reasons, three different products obtained from the skins, seeds and the whole pomace (skin and seeds) of wild blueberries (*Vaccinium myrtillus* L.) have been characterised in this work. The proximate, mineral, anthocyanin, phenolic and antioxidant content of the three products was evaluated, together with a microbial characterization. Furthermore, the effect of heat treatment at 90 °C for different periods of time (30, 60, 90 and 120 min) on the anthocyanin and phenolic profiles, as well as the microbiological stability of the products, was investigated to ensure their safety. The heat treatment of 90 °C was selected according to the results obtained in a previous work in which two different treatments, heat treatment and UV treatment, were applied to powdered products obtained from grape pomace in order to reach their microbiological stabilization. The best results were obtained with a heat treatment at 90 °C [28].

## 2. Materials and Methods

### 2.1. Chemicals

The pure phenolic compound standards quercetin, quercetin-3-O-glucoside and delphinidin-3-galactoside were purchased from Extrasynthese (Genay, France); delphinidin-3-glucoside, cyanidin-3-glucoside, cyanidin-3-galactoside, cyanidin-3-arabinoside, petunidin-3-glucoside, peonidin-3-glucoside and malvidin-3-glucoside were supplied by Polyphenols (Sandness, Norway); and caffeic acid, chlorogenic acid, p-coumaric acid, gallic acid, methyl gallate, protocatechuic acid, synaptic acid, and syringic acid were supplied by Sigma-Aldrich-Merck (KGaA, Darmstadt, Germany). An ICP multielement standard IV solution from Merck (KGaA, Darmstadt, Germany) was employed for the quantification of the minerals. Regarding solvents, acetonitrile (≥99.9%), acetonitrile HPLC-MS, acetic acid, formic acid LC-MS and methanol were supplied by VWR International (Barcelona, Spain). Deionised water was obtained using a water system Milli-Q Q-POD^®^ Integral 3 from Merck Millipore (Darmstadt, Germany). Nitric acid (65%) and the hydrogen peroxide (30%) suprapure quality were from Supelco, and formic acid (98–100%) was from EMSURE^®^, with all of them supplied by Sigma-Aldrich-Merck (KGaA, Darmstadt, Germany). Unless stated otherwise, all other chemicals and reagents were obtained from Sigma-Aldrich-Merck (KGaA, Darmstadt, Germany) or Fisher Scientific (Loughborough, UK).

### 2.2. Samples

**Figure 1:**
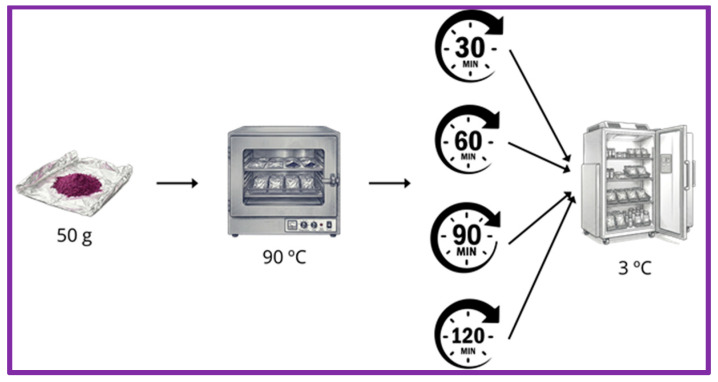
Diagram of the thermal treatment applied.

The pomace (solid residue mixture of skins and seeds) resulting from blueberry juice processing was the raw material used in this study. It was supplied by a local supplier. This waste had an initial moisture of 40%. The pomace was spread in a thin layer on a tray and was dried in a hot air oven at 60 °C for 3 h (Serie 2000, Selecta, Barcelona, Spain) to achieve a final moisture lower than 5%. From dried blueberry pomace, three different products were obtained, one from the whole pomace, labelled as Sk + S (skins + seeds), and two from the corresponding main constituent of the pomace, the skins, labelled as Sk, and the seeds labelled as S, which were separated by sieving (0.8 mm). To obtain the final products, the corresponding raw material, whole pomace, skins and seeds, was milled and sieved. Subsequently, the seeds were processed in a coffee grinder to obtain a final powdered product with a particle size of less than 0.5 mm, while skins and the whole blueberry pomace were ground in a hammer mill (Pulverisette, Dietz, Fritsch, Germany) and sieved to keep the fraction of less than 0.35 mm. Thermal treatments at 90 °C at different times (30, 60, 90 and 120 min) were tested in the final powdered products to reach the optimum microbial inactivation and to study the effect of the heat treatment on the phenolic composition of the samples. The thermal treatment was carried out in a hot air oven (Serie 2000, Selecta, Barcelona, Spain). Fifty grams of blueberry product were wrapped in aluminium foil. Then, the packages were placed on the oven tray. After drying, the samples were vacuum-packed in cast polyamide/polyethylene (Vacioplast Salamanca, Salamanca, Spain) and stored at 3 °C until analysis. A diagram of the heat treatment applied is shown in Figure 1.

### 2.3. Main Composition Analysis

The moisture content of the three dried samples was determined by measuring their weight before and after drying in an oven at 105 °C for 6 h (Selecta). To determine the pH, 50 mL of water were added to 2.5 g of the sample. The samples were stirred for 1 h at 35 °C, after which the pH was measured using a pH meter (ORP Sensor+ pH3, Hach, Düsseldorf, Germany). Acidity was determined by adding 0.1 N sodium hydroxide until the pH reached 7, using the same sample that was used to evaluate the pH. The results were expressed as g of citric acid per g of sample. Protein content was determined by digesting the samples with sulphuric acid and measuring the nitrogen content using the Kjeldahl method. The Soxhlet method (B-811, BUCHI, New Castle, NE, USA) was used to determine the fat content with petroleum ether as the solvent.

The reducing sugars present in the products were extracted as follows: 1 g of the sample was weighed into a centrifuge tube, to which 20 mL of distilled water was added. The tubes containing the samples were shaken continuously for 24 h on an orbital shaker (Rotabit, Selecta, Barcelona, Spain). The samples were then centrifuged at 8000 rpm for 10 min at 20 °C in a centrifuge (Centrikon T-124, Kontron Instruments, Deggendorf, Germany). The supernatant was then filtered through a cellulose filter (Ø 125 mm, 8–12 µm pore size, (VWR International, Barcelona, Spain) and brought up to a final volume of 25 mL. Then, in a 250 mL Erlenmeyer flask, 10 mL of a 0.26 M CuSO_4_ solution, 5 mL of Seignette salt (kNaC_4_H_4_O_6_-4H_2_O) solution and 2 mL of the sample were added. This mixture was boiled for 3 min and then immediately cooled. Then, 10 mL of 1.8 M KI, 10 mL of 16% H_2_SO_4_ solution and 10 mL of starch indicator were added successively, stirring continuously. The solution was then titrated with 0.1 N Na_2_S_2_O_3_ until a milky white final colour was obtained, corresponding to V1 = mL of sodium thiosulfate used. A blank was made by replacing the sample with distilled water. The volume of Na_2_S_2_O_3_ used in the blank titration corresponds to V2. V1–V2 indicates the g/L of reducing sugars present in the sample. Total dietary fibre was determined using the enzymatic-gravimetric method (AOAC Method 985.29 and 960.52, 1997) with the enzymatic kit supplied by Sigma-Aldrich (TDF 100-1KT, Merck KGaA, Darmstadt, Germany).

### 2.4. Mineral Content

The minerals were determined using Optical Emission Spectrometry (ICP-OES) with a SPECTRO Arco II spectrometer (SPECTRO Analytical Instruments, Kleve, Germany). The samples were previously digested using an Ethos SEL microwave (Milestone, Sorisole, Italy), with 0.15 g of sample introduced into the digestion tubes. Then, 8 mL of nitric acid and 2 mL of 30% H_2_O_2_ were added to the samples. The following temperature ramp was applied: room temperature to 80 °C in 4 min, 80 °C to 120 °C in 4 min and 120 °C to 170 °C in 5 min. Finally, 170 °C was held for 30 min. The following inorganic elements were quantified: K, Ca, Na, P, Fe, Mg, Mn, Se and Zn. The linear correlation coefficients of the calibration curves for the analysed elements were all above 0.99.

### 2.5. Extractable Main Phenolic Compounds and Associated Antioxidant Capacity

To determine the extractable anthocyanins, total phenolic compounds and corresponding antioxidant activity, a solid–liquid extraction was carried out on each powdered product. Then, 10 mL of a methanol:formic acid:distilled water mixture (80:1.5:18.5 *v*/*v*) was added to 1 g of a sample that previously had been hydrated with 3 mL of water. The mixture was then sonicated for 10 min using a 50 Hz ultrasonic generator (JP Selecta, Barcelona, Spain). The samples were then centrifuged at 8000 rpm for 10 min at 20 °C (Centrikon T-124), after which the supernatant was separated from the solid by decanting. This extraction process was repeated three times. The four resulting extracts were pooled and brought up to a volume of 50 mL with the same solvent. Three replicates were prepared for each powdered product (Sk, S and Sk + S).

To determine total anthocyanin content, the method described by Paronetto [29] was followed. It is based on the property of anthocyanins to change colour as the pH of the medium varies. Two test tubes were prepared as follows: tube A (sample), to which 1 mL of extract and 10 mL of 1 N HCl were added; and tube B (blank), to which 1 mL of sample and 10 mL of phosphate-citrate buffer (pH 3.5) were added. The tubes were shaken and the absorbance of the sample at 525 nm was measured immediately against the blank in a spectrofotometer (U-I900, Hitachi, Tokyo, Japan). For the quantification of anthocyanins, the absorbance measurements were plotted on a calibration curve to obtain the anthocyanin concentration expressed in mg/L of cyanidin-3-O-glucoside. The calibration curve was prepared from a stock solution of 500 mg/L cyanidin-3-O-glucoside, and solutions ranging from 25 to 250 mg/L were prepared. The final results was expressed as mg cyanidin-3-O-glucoside/g.

The method described by Singleton and Rossi [30] was followed for the determination of total polyphenols. This method is based on the oxidation, in a basic medium, of the hydroxyl groups of phenolic compounds by Folin–Ciocalteu reagent, resulting in a blue-coloured compound that can be measured spectrophotometrically at 750 nm. To determine total polyphenols, 0.5 mL of sample, 0.5 mL of Folin–Ciocalteu reagent and 10 mL of CaCO_3_ (75 g/L) were added to a 25 mL flask. The mixture was shaken and made up to 25 mL with distilled water. It was left to stand for 1 h in the dark. In addition, a blank was prepared under the same conditions. Finally, the absorbance of the sample at 750 nm was measured against the blank in a spectrofotometer (U-I900, Hitachi, Tokyo, Japan). Quantification was carried out using a calibration curve, where the absorbance measurement at 750 nm was interpolated, thus obtaining the total polyphenol concentration expressed in mg/L of gallic acid. The calibration curve was prepared from a stock solution of 500 mg/L gallic acid, and solutions ranging from 25 to 500 mg/L were prepared. The final results was expressed as mg gallic acid/g.

The ABTS method determines the antioxidant capacity of a sample. It is based on the decolourisation of the ABTS^•+^ radical (2,2′-azinobis-(3-ethylbenzothiazoline-6-sulfonic acid)), which is generated by the addition of an oxidising agent, K_2_S_2_O_8_. This radical exhibits a maximum spectrophotometric absorption at 734 nm. In the presence of an antioxidant agent, the compound decolourises, resulting in a decrease in absorption. The ABTS assay was performed according to the procedure of Rivero-Pérez et al. [31] with minor modifications. The ABTS^•+^ cation radical was prepared by mixing equal parts of ABTS reagent (7 mM) and 2.45 mM K_2_S_2_O_8_. The mixture was kept in the dark for at least 16 h to ensure that the reaction had been completed. After this time, the mixture was diluted with milli-Q water until an absorbance of between 0.7 and 0.9 was achieved at 734 nm; this absorbance was taken as the initial absorbance. Next, 20 µL of the extract and 980 µL of the diluted ABTS reagent were added to a 1.5 mL Eppendorf tube. After waiting 15 min in the dark, the absorbance was measured at 734 nm using a spectrophotometer (U-I900, Hitachi, Tokyo, Japan). The antioxidant activity of the sample was calculated as the difference between the initial and final absorbances. The difference was plotted on a calibration curve obtained from a 10 mM Trolox standard solution, from which solutions ranging from 0.1 mM to 1.4 mM were prepared. The results were expressed in mmol Trolox/g.

### 2.6. Microbial Load and Microbiological Stability

As the ultimate goal is to use the three products obtained from the blueberry pomace in foodstuffs, it was necessary to evaluate and characterise their initial microbiological state.

The following microbiological parameters were quantified for each of the prepared products (Sk + S, Sk and S): aerobic mesophilic bacteria (AMB), *Enterobacteriaceae*, Lactic Acid Bacteria (LAB), *Bacillus* spp., *Clostridium* spp., moulds and yeasts. To achieve this, 1 g of each product was weighed under aseptic conditions and 13 mL of Brain Heart Infusion broth (BHI, OXOID, Basingstoke, UK) was added. Serial decimal dilutions were then prepared from this dilution in buffered peptone water (Condalab, Torrejón de Ardoz, Spain). To quantify AMB and *Enterobacteriaceae*, 1 mL of the corresponding dilutions was inoculated using the pour-plate method onto plate count agar (PCA, Condalab) and violet red bile agar with glucose (VRBG, Condalab) with a double layer, respectively. To quantify LAB, *Bacillus* spp., *Clostridium* spp., moulds, and yeasts, 100 µL of the dilutions were streaked onto M.R.S. agar (Oxoid), M.Y.P. base agar (Oxoid) supplemented with sterile egg yolk (Oxoid) and polymyxin B supplement (Oxoid), Tryptone sulfite neomycin agar (TSN, Condalab), and Sabouraud chloramphenicol agar (Scharlau, Barcelona, Spain), respectively. Once inoculated, agar plates were incubated at 30 °C for 48 h (AMB and LAB); 30 °C for 5 days (moulds and yeasts); 30 °C for 24 h (*Bacillus* spp.); 37 °C for 24 h (*Enterobacteriaceae*); and 37 °C for 24 h under anaerobic conditions generated with an AnaeroGen sachet (OXOID) (*Clostridium* spp.).

### 2.7. HPLC-QTOF Analysis

Identification of anthocyanins and phenolic compounds was based mainly on their mass spectra. A HPLC equipment (1260 infinity, Agilent Technologies, Santa Clara, CA, USA) fitted to a Time of Fly (Q-TOF) detector mass spectrometer (6545, Agilent Technologies, Santa Clara, CA, USA) equipped with an electrospray ionization (ESI) source, and positive electrospray ionization mode, was used for data acquisition. Anthocyanins analysis was carried out with a Nova-Pak^®^, C18 4 µm, 3.9 × 300 mm column (Waters, Chromatografía S. A., Cerdanyola del Vallès, Barcelona, Spain). The chromatographic conditions were as follows: flow, 0.7 mL/min; injection volume 15 µL; mobile phases: A: water: formic acid (99:1 *v*/*v*); B: methanol: formic acid (99:1 *v*/*v*). The gradient used was: 0–0.5 min: A (100%); 0.5–26 min: A (75%); 26–40 min: A (50%); 40–55 min: A (30%). The source parameters were sheath gas temperature 350 °C; gas flow, 10 mL/min; nebulizer pressure, 50 psi; skimmer: 60, nozzle voltage 500 V; fragmentor 100; Vcap: 3500. The mass spectrometer was set to acquire over *m*/*z* 70–1000. For the identification of the phenolic compounds negative electrospray ionization mode was used for data acquisition. The column employed was a Spherisorb^®^ 3.0 µm ODS2, 4.6 mm × 250 mm (Waters, Chromatografía S. A.). The chromatographic conditions were as follows: flow 0.70 mL/min; injection volume 50 µL; mobile phases: A: water: formic acid 0.5%; B: water: acetonitrile: formic acid (79:20.5:0.5). C: acetonitrile: formic acid (95:5) The solvent gradient used was: 0–0.5 min: A (100%); 0–26 min: A (75%), B (25%); 40–55 min: A (50%), B (50%); 55–99 min: A (30%), B (70%); 99–100 min: B (100%); 100–118 min: B (70%) C (30%); 118–123 min: B (40%) C (60%); 123–148 min: C (100%); 148–155 min: C (100%). The following source parameters were modified with respect to the method employed for anthocyanin identification: sheath gas temperature 320 °C, gas flow 8 mL/min. Fragmentor 165. The mass spectrometer was set to acquire over *m*/*z* 70–1100.

### 2.8. HPCL-DAD Analysis

The quantification of anthocyanins and phenolic compounds was carried out by HPLC-DAD employing a 1200 series Agilent HPLC (Agilent Technologies) coupled to a Diode Array Detector of the same model and supplier. The chromatographic separation of anthocyanins was carried out on the same reverse phase Nova-Pak C18 column used for the HPLC-QTOF analysis. The solvents used were (A) water/formic acid (90:10) and (B) methanol/water/acetonitrile/formic acid (40:40:10:10). The gradient used was the following: 0–20 min: A (75%); 20–25 min: A (75%); 25–35 min: A (35%). Detection was carried out in scan mode from 250 to 600 nm and quantification at 530 nm. Calibration curves from the respective standards, when available, were used for quantification. The available standards (delphinidin-3-galactoside, delphinidin-3-glucoside, cyanidin-3-galactoside, cyanidin-3-glucoside, cyanidin-3-arabinoside, petunidin-3-glucoside, peonidin-3-glucoside and malvidin-3-glucoside) were used to quantify the respective anthocyanins, while the others were quantified as equivalent of their respective glucoside. So, for example, petunidin-3-galactoside and petunidin-3-arabinoside were quantified using the calibration curve for petunidin-3-glucoside, and the same approach was used for the other cases.

The same column used for identifying the phenolic compounds by HPLC-QTOF was used for quantifying them. The chromatographic conditions were as follows: flow: 0.7 mL/min. Injection volume: 100 µL. Mobile phases: A: water and glacial acetic acid (98:2, *v*/*v*); B: water, acetonitrile and glacial acetic acid (78:20:2, *v*/*v*/*v*); and C: acetonitrile. The same solvent gradient was used as in the HPLC-QTOF analysis. The eluent was monitored at 254, 280, 320, 360 and 520 nm, and compound spectra were obtained between 240 and 600 nm. Phenolic acids and catechin derivatives were quantified using calibration curves obtained from their respective standards. All flavonols were quantified as quercetin-3-glucoside equivalents.

The different methanolic extracts were diluted with Milli-Q water (1:4) and filtered through 0.45 µm pore size PVDF filters (Teknokroma Analítica S.A., Sant Cugat del Vallès, Barcelona, Spain) before being injected into the HPLC. All results were expressed in mg/g of product.

### 2.9. Statistical Analysis

For the statistical treatment of the results, a simple analysis of variance (ANOVA) was performed to determine factor effect (temperature or type of product). In addition, Fisher’s LSD (Least Significant Difference) test with a confidence level of 95% (*p*-value < 0.05) was applied to determine the significant differences between samples. Statgraphics Centurion 19 X64 software was used.

## 3. Results and Discussion

### 3.1. Characterization of the Powdered Products

The dry Sk + S and Sk products had a similar proximate composition. However, some statistically significant differences were found between these products and those obtained from the seeds (Table 1). The lack of statistically significant differences between the Sk + S and Sk products may be because the seeds accounted for only 3% of the total blueberry pomace. Furthermore, due to their small size and thickness, the seeds were more difficult to break into small particles with the mill used. During the sieving process, some of the seeds could be retained and did not pass through the sieve with a mesh size of 0.350 mm, which was used for the study.

Total dietary fibre was the main component of the three products studied, with the S product having the lowest values. According to Struck et al. [32], dietary fibre constitutes approximately 68% of dried blueberry pomace, which is consistent with the results found in this study. Aura et al. [33] also found fibre concentrations of 58.9% in bilberry press cake. However, these concentrations are much higher than those reported by other researchers. For instance, Calabuing-Jiménez et al. [21] found total fibre concentrations of 34–38% in *Vaccinium corymbosum* powder, while Tagliani et al. [34] found fibre concentrations of 26% in dried O’Neill variety blueberry pomace. The high fibre content of blueberry pomace is due mainly to dietary fibre (60.8%) that is largely insoluble (46.2% vs. 14.6% soluble dietary fibre), pectin, xyloglucan, arabinoxylan and mannan polysaccharides [35].

Lipids were the second main component in the studied products, with the seed product having the highest value (21%). According to the published results seeds are rich in polyunsaturated fatty acids (linoleic, linolenic and oleic acids) [36]. However, the values obtained for the S product in this study are slightly lower than the fat content found in highbush and Rabbiteye blueberries (24.91% and 26.90%, respectively) [37]. Meanwhile, the fat content in the Sk and Sk + S products is higher than the 4.1% found in O’Neill cultivar blueberry pomace powder [34] and the 2.45% and 2.59% found in highbush and Rabbiteye blueberry peels, respectively [36].

The seed product also presented the highest protein values compared to the Sk + S and Sk products. The protein concentrations found in this study were higher than those found in the blueberry pomace product (6.6%) obtained by Tagliani et al., [34] and in the blueberry skins and seeds (0.8–7.8%, respectively) studied by Li et al. [36]. Apart from the effects of variety and growing area, the differences observed in the fat and protein results in this study compared to those reported in the literature could be related to differences in carbohydrate composition, since the results are expressed as a percentage. Reference [34] and [36] found carbohydrate contents of 60.9% and 44.04–50.06%, respectively. These values are much higher than the values found for reducing sugars in this study (see Table 1).

These differences in sugar concentration may be related to differences in the method employed to obtain the pomace products, and in the analytical method employed for the carbohydrate determination.

### 3.2. Mineral Content Results

The three products had a high P, Ca and K content, and were also rich in Mg, Fe and Mn. Other essential minerals, such as Zn and Cu, were present in lower amounts (Table 2). Minerals are vital nutrients essential for maintaining human health by modulating a wide range of physiological functions. They contribute fundamentally to bone development, enzyme activation, nerve signaling, immune response, and cardiovascular and metabolic regulation [37]. Determining their levels in products derived from blueberry pomace is of great interest in view of their potential use as functional ingredients, as they could be used in the production of mineral-enriched foods. The amount of Na found in the products obtained, particularly those from whole pomace and skins was low, which is favourable from a health perspective due to its association with hypertension. To the best of the authors’ knowledge, no data has been published on the mineral composition of blueberry pomace products. However, various studies have indicated that blueberries are a significant source of P, K, Ca, Mg and other trace elements such as Mn, Zn and Cu [4,5,6]. Krstić et al. [38] studied the mineral composition of cultivated blueberry seeds. They found that Ca and P were the most abundant elements, followed by K and Na. The amounts of these minerals found by these authors in blueberry seeds are higher than those found in the present study. These differences could be related to the effect of variety and region of origin on the mineral composition of berries [6,39,40].

### 3.3. Total Anthocyanins, Total Phenolics and ABTS of Non-Thermally Treated Samples

**Figure 2:**
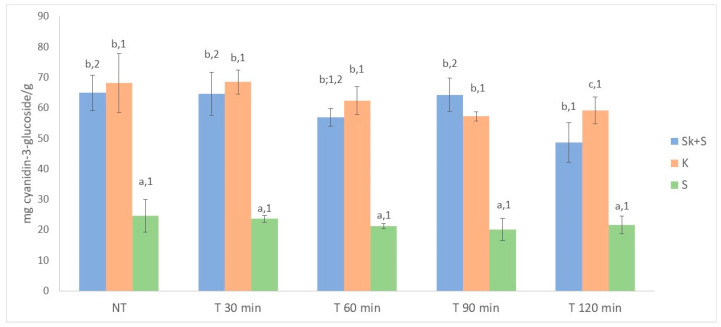
Effect of heat treatment on total anthocyanin content. Different letters denote significant differences (LSD test and *p* < 0.05) between products. Different numbers denote significant differences, (LSD test and *p* < 0.05) between thermal treatments. Sk + S: product obtained from the whole pomace (skins and seeds); Sk: product obtained from skins; S: product obtained from seeds NT: no thermal treatment.

**Figure 3:**
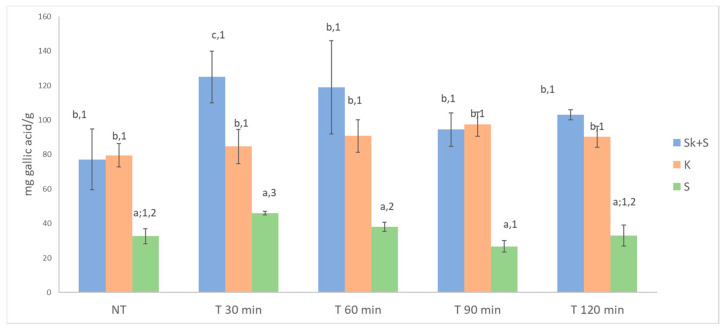
Effect of heat treatment on total polyphenolic content. Different letters denote significant differences (LSD test and *p* < 0.05) between products. Different numbers denote significant differences, (LSD test and *p* < 0.05) between thermal treatments. Sk + S: product obtained from the whole pomace (skins and seeds); Sk: product obtained from skins; S: product obtained from seeds NT: no thermal treatment.

**Figure 4:**
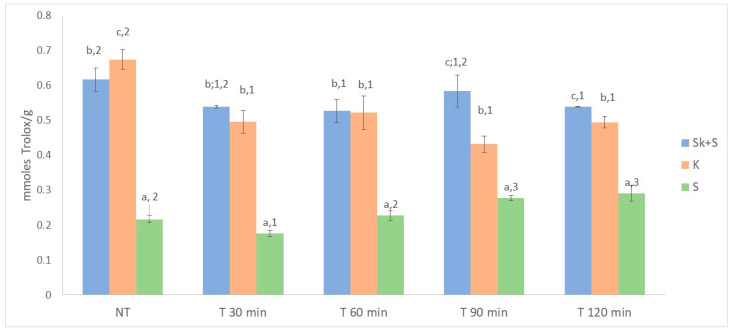
Effect of heat treatment on the antioxidant capacity evaluated following the ABTS method. Different letters denote significant differences (LSD test and *p* < 0.05) between products. Different numbers denote significant differences, (LSD test and *p* < 0.05) between thermal treatments. Sk + S: product obtained from the whole pomace (skins and seeds); Sk: product obtained from skins; S: product obtained from seeds; NT: no thermal treatment.

Anthocyanins are responsible for the black, blue and red pigments in berries, including blueberries. The concentration and composition of anthocyanins are important for both the sensory quality of fruits and derivate products, as well as their health benefits [41]. The extractable anthocyanin content of the Sk and Sk + S products was similar and much higher than that of the S product, being almost three times higher (Figure 2). This is because anthocyanins are found in blueberry skins, but not in seeds [42,43]. The extractable anthocyanin values found in the S product are due to anthocyanins that have been absorbed onto the surface of the seeds as well as remnants of skins attached to the seeds that could not be separated adequately by sieving. The S products had around half the concentration of extractable total phenolic compounds of the other two products (Sk + S and Sk), which is consistent with their higher extractable anthocyanin content. No data has been found regarding the anthocyanin and phenolic content of blueberry seeds (Figure 3). However, the levels of total anthocyanins and total polyphenols found in the products obtained from the skins were similar to those reported [44]. Nemetz et al. [45] also observed similar concentrations of total anthocyanins and total phenolic compounds in bilberry pomace powder, although slightly lower (25 and 30 mg/g respectively). On the other side, the levels reported by other authors in blueberry pomace are much lower than those found in this work. Thus [46] found concentrations of total anthocyanins and total polyphenols of 1.07–2.58 mg/g and 2.47–8.5 mg/g respectively, whereas [47] found concentrations of 0.377–0.420 mg/g of total anthocyanins and 5.3–6.8 mg/g of total polyphenols. Corresponding to the higher levels of extractable anthocyanins and total polyphenols of the Sk and Sk + S products, higher antioxidant capacity values were found in their extracts compared to those from the seed products (Figure 4). This is likely because the antioxidant activity of blueberries is directly related to the content of phenolic compounds [41]. Tagliani et al. [34] and Baenas et al. [48] observed similar results (0.339 mM/Trolox/g and 0.278 mM/Trolox/g, respectively) to those found in this work, although they worked with other blueberry cultivars. However, other authors found lower levels of antioxidant activity, despite using the same method of evaluation [21,46]. Different works have highlighted the effect of the method used to prepare the juice, the cultivar origin, growth environment, genetics and cultivation, which can have a significant impact on the final concentrations of anthocyanins and phenolic compounds in the by-products generated [44,49]. These factors explain the high variability in results reported in the literature.

### 3.4. Identification and Quantification of Anthocyanidin Compounds

The anthocyanins detected in the powdered products obtained from the blueberry pomace were identified by considering the *m*/*z* signal and fragment ion data, the retention times of the reference standards (when available) and their UV-Vis spectra. The results were also contrasted with those reported in the literature [10,46,50,51,52]. Multiple identification tools must be used to confirm the identity of anthocyanins without standards due to their high degree of structural complexity and similarity, since results from accurate mass measurements alone are insufficient to report a new natural product even though such practice is sometimes found in the literature [49]. HPLC-ESI-MS and MS/MS analyses were performed in positive ion mode only, as these compounds are present as flavylium ions under the applied chromatographic conditions and therefore easily produce molecular cations [M]^+^ in their ESI mass spectra [53]. The average mass accuracy of the measurements was within 5 ppm (Table 3). The UV-Vis spectrum of the anthocyanin quantified are shown in Supplementary Materials (Figure S1).

Eighteen anthocyanins were identified in the three products (Table 4). Most of them had previously been detected in blueberries [50,52,54]. All of them were glycosylated derivatives of cyanidin, delphinidin, malvidin, peonidin and petunidin, (arabinosides, galactosides and glucosides); three of the anthocyanins detected were also acylated anthocyanins with p-coumaric acid esterified at the 6th carbon of the glucose molecule. The latter were present at very low levels, which are insignificant compared to non-acylated anthocyanins (Table 4). Nevertheless, their presence is noteworthy as, to the best of the authors’ knowledge, this is only the second time that these types of anthocyanins have been described in blueberries. While Dong et al. [55] detected two p-coumaroyl glucosides (malvidin and petunidin), this paper reports the detection of three p-coumaroyl glucosides (cyanidin, delphinidin and malvidin). Regarding the sugar link to the aglycone, the data obtained are consistent with those published previously, which point out that L-galactose and mainly D-glucose are the most abundant sugars associated with anthocyanins in wild and cultivated *Vaccinium* species, and that arabinose is the most abundant pentose sugar associated with flavonoids in these berries [50,52]. In agreement with total anthocyanins contents, seed products showed significantly lower quantities of individual anthocyanins, and Sk and Sk + S products showed quite similar contents (Table 4). Based on the quantification data, delphinidin-3-glucoside, which has been reported to be responsible for the blue colour of berries such as blueberries, and petunidin-3-glucoside appeared to be the two main anthocyanins (Table 4) [10,56]. Lätti et al. [57] studied the growth of blueberries (*Vaccinium myrtillus*) in different regions of Finland and observed that the dominant aglycone in the northern area was delphinidin, whereas cyanidin prevailed in the central and southern areas. Buchert et al. [54] identified delphinidin-3-arabinose and delphinidin-3-galactoside as the major anthocyanins in Finnish blueberries. However, ref. [10,51] reported cyanidin-3-glycosides as the major anthocyanins in this species. Other studies carried out with different blueberry species [49,52,58] found that the main anthocyanidin was malvidin, followed by delphinidin and petunidin. Therefore, the results of these studies showed remarkable variation in the anthocyanin composition of different blueberry species, cultivars and populations. This is because the composition of anthocyanins is strongly influenced by numerous factors, both intrinsic and extrinsic to the fruit. These factors include the species, the size of the fruit, the degree of ripeness, and the environmental conditions to which the fruit is exposed during harvest and storage [59].

### 3.5. Identification and Quantification of Phenolic Compounds

A total of 18 phenolic compounds were identified (Table 5), seven phenolic acids and two derivatives; seven flavonols; and two flavan-3-ols. All of these compounds have previously been described in the literature as being present in blueberries [10]. The UV-Vis spectrum, molecular ion fragmentation pattern in MS/MS and retention time of standards were used for assessment when available (Table 6). Furthermore, the results were compared with those found in the literature [10,60,61,62]. The UV-Vis spectrum of the phenolic compounds quantified are shown in Supplementary Materials (Figure S2).

Quantitative data indicated significant differences between the levels of the phenolic compounds identified. As for anthocyanins, the Sk and Sk + S products showed higher concentrations of the compounds studied than the seeds product. Syringic, sinapic and chlorogenic were the phenolic acids present at the highest concentration in the three products. They have also been found in blueberries by others authors [61,63]. In the products studied, the p-coumaric, caffeic and chlorogenic acids, together with the associated phenolic (p-coumaric acid hexoside), represent between the 29% and 37% of the acids and derivatives quantified. The seed product presented higher percentages of these compounds which is correlated with the lignin biosynthesis [64]. Most of the flavonols found in blueberries are glycosylated, mainly with galactose, glucose, and rhamnose. Other less common glycosides, such as arabinosides, xylosides, acetylated diglycosides and glucuronides, have also been reported [62,65]. The glycosylated flavonols quantified in this study were only partially identified (Table 6). This was because it was not possible to determine the sugar linked to the corresponding aglucone. The reason behind this is that the fragments generated by MS/MS were the same for glucose and galactose, and for xylose and arabinoside, and no standards were available. The two quercetin hexosides were the major flavonols in the three products, which agrees with the results found in different species and cultivars of blueberries [60,62,65]. Proanthocyanidin B2 and epicatechin were the two flavan-3-ols identified in the three studied products. Both have been reported previously in blueberries [41,61], although the concentrations of epicatechin were significantly lower than those found in wild bilberry pomace (0.85 mg/g of sample) [66].

Some of these phenolic compounds are also known to be astringent, especially myricetin-3-O-arabinoside, myricetin-3-O-glucoside, myricetin-3-O-galactoside and an unknown caffeoyl quinic acid [42].

### 3.6. Microbiological Results

The microbiological load of the powered products obtained from the blueberry pomace was studied in order to know the possibility of its future application in functional foods without compromising the safety and quality of the food to which it will be added.

Data of the microbiological load of the studied products before their thermal treatment (Table 7) indicated that the total load of AMB in the seeds was higher than in the case of the whole product (Sk + S) or the skins alone and that the spore forming *Bacillus* spp. was present in the three products. *Clostridium* spp. was not quantified in any product, while *Enterobacteriaceae* and LAB were only quantified in the S and Sk + S, respectively. Moreover, moulds and yeast were not quantified in the S product. In general, the load for each microbial parameter is low, except for AMB and *Enterobacteriaceae* in the case of S product. Reißner et al. [67] reported a range of 3.2–5.8 cfu/g of viable counts and 2.1–3.1 cfu/g in different dried berry pomaces (moisture content less than 5%), results higher than those found in this study. Higher loads of viable counts (2.0 × 10^5^ cfu/g) and moulds (5.0 × 10^3^ cfu/g) were found in grape seeds flour naturally dried with 4.1% moisture content [68]. Torrefacto silverskin oven-drying at 40 °C for 24 h (7.89% moisture content) presented similar AMT concentration (8.3 × 10^3^ cfu/g) to the blueberry powered seeds product [69]; however, no information has been found on dried blueberry pomace.

### 3.7. Effect of the Thermal Treatment of Products

#### 3.7.1. Modifications on Microbiota

**Figure 5:**
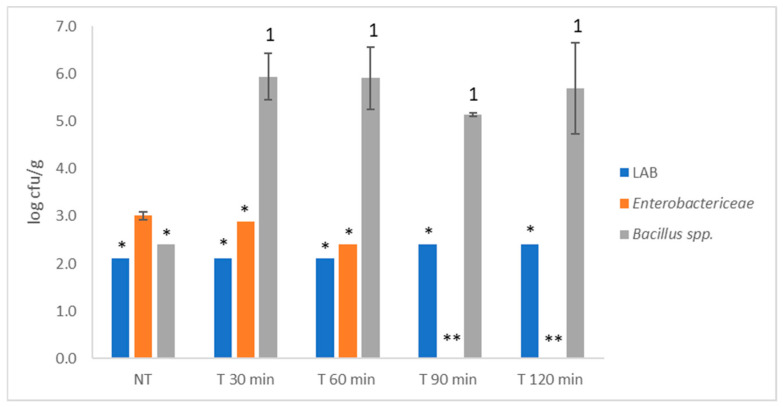
Effect of heat treatment (90 °C) on the lactic acid bacteria (LAB), *Enterobacteriaceae* and *Bacillus* spp. of the seed-powered product. Different numbers denote significant differences (LSD test and *p* < 0.05) between treatments. NT: no thermal treatment. * denote that one replicate was below the detection limit (2.11 lof cfu/g for LAB and *Bacilllus* spp. and 1.11 log cfu/g for *Enterobacteriaceae*); ** denote both replicates were below the detection limit (1.11 log cfu/g for *Enterobacteriaceae*).

To improve the microbiological quality of the products by reducing or inactivating the microbiota present, the dried powdered products obtained were subjected to a heat treatment of 90 °C for 30, 60, 90 and 120 min. After the four heat treatments, both Sk + S and Sk products exhibited a significant improvement in microbiological quality, as only AMB was detected. In contrast, the microbiological quality of the seed product did not improve (Figure 5); the initial load of LAB persisted after all heat treatments, and similar levels of initial load of *Enterobacteriaceae* were observed after 30 and 60 min. Additionally, the heat treatment increased the number of *Bacillus* spp. by more than 5 log cfu/g after every treatment and no differences were observed between treatments (*p* > 0.05).

**Figure 6:**
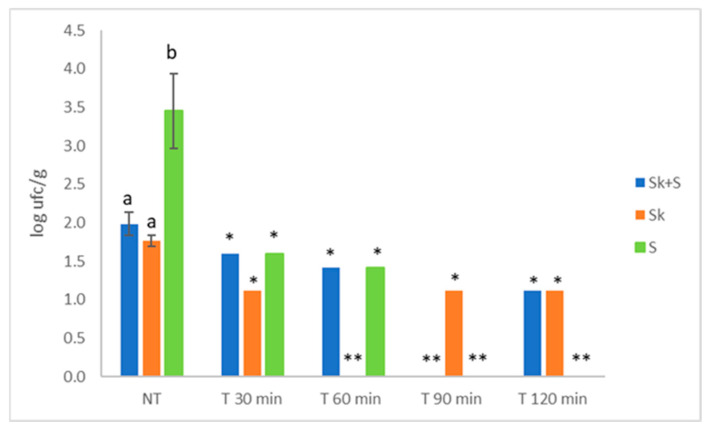
Effect of heat treatment (90 °C) on the aerobic mesophilic bacteria (AMB) of the pomace powdered products. Different letters denote significant differences (LSD test and *p* < 0.05) between products. Sk + S: product obtained from the whole pomace (skins and seeds); Sk: product obtained from skins; S: product obtained from seeds; NT: no thermal treatment; * denote that one replicate was below the detection limit (1.11 log cfu/g); ** denote both replicates were below the detection limit (1.11 log cfu/g).

The most effective treatment for reducing the AMB load was 90 °C for 90 min (Figure 6), as it reduced its concentration below the quantification limit in the Sk + S and S product, being the S product the most contaminated initially. This treatment was also effective for similar products obtained from grape pomace, reducing AMB, mould, and yeast concentrations below the detection limit in the product derived from grape seeds [28]. A heat treatment of 65 °C during 30 min was able to reduce below the detection limit (30 cfu/g) the initial content (4.4 × 10^3^ cfu/g) of total counts of microorganisms incubated at 22 °C; however, the moisture content was 73.1% which may favoured the inactivation of the microorganisms [70]. Although the studied products cannot be classified within the group of spices and dried culinary herbs, the code of hygienic practice for low-moisture foods recommend the absence of *Salmonella* and *E. coli* (*Enterobacteriaceae*), *Clostridium* spp., *Bacillus cereus* and mycotoxin-producing moulds [71], a situation observed in the case of Sk + S and Sk but not in S products. Moreover, the first two products also met the microbiological criteria established in the European Union (EU) for cranberry extract powder as a novel food [72].

#### 3.7.2. Effect of the Heat Treatment on the Anthocyanin and Phenolic Compounds

The data on total phenolic compounds (Figure 3) showed that thermal treatment generally had no significant effect on the amount of extractable phenolic compounds. A significant effect of temperature was only observed for the seed products. However, the results did not reveal a clear effect of treatment time on the content of phenolic compounds extracted. As for total anthocyanin content, statistically significant differences in total extractable anthocyanins were only found in the Sk + S product, with the 120 min treatment yielding the lowest anthocyanin values (Figure 2). It is well-established that heat treatment decreases the concentration of anthocyanins in berries due to degradation [3,25,26]. In this regard, Martín-Gómez et al. [58] reported an increase in the anthocyanin concentration of blueberries (*Vaccinium corymbosum*) during the drying process. However, the highest temperature studied by these authors was 50 °C. Cesa et al. [73] reported that the total anthocyanin content of blueberry purées was about 5% higher after rapid pasteurisation at 70 °C for 15 s than in unpasteurised samples. This confirms that this treatment is capable to inactivate the polyphenol oxidase involved in anthocyanin degradation, thus preserving these bioactive compounds. Instead, when blueberry purées were thermally stressed at 70 °C for 2 h, losses of anthocyanins exceeding 25% were observed, indicating that, under prolonged pasteurisation conditions, thermal degradation of anthocyanins prevails over their preservation. Regarding the effect of heat treatment on antioxidant capacity, significant differences were observed in the three products studied (Figure 4). The heat-treated Sk + S and S products showed lower ABTS values than the untreated ones. However, an increase in antioxidant activity was observed in the treated seed products after 90 min of treatment. Kim et al. [74] also observed an increase in the antioxidant activity of powdered grape seed extracts after heating. This could be due to different reasons, such as the transformation of some phenolic compounds into others by hydroxylation and epimerisation [75], or the release of phytochemicals from the cell matrix due to heat treatment, which makes them more accessible [74,75,76]. Furthermore, the formation of novel compounds, such as Maillard products, could be associated with the increase in antioxidant activity of products from thermal processes [76].

Considering the thermolability of anthocyanins, it was considered interesting to evaluate the effect of thermal treatment on the levels of the individual anthocyanins. The results showed that, in general, treatment for 120 min significantly decreased the levels of individual anthocyanins (Table 4). Shorter thermal treatments did not clearly affect the anthocyanin content; only some of them increased or reduced its concentration due to the heating. This effect may be related to intermolecular interactions between anthocyanins and different components of the matrix such as polysaccharides and sugars that stabilize anthocyanins by absorbing the flavylium cation [55,77] and/or to inter and intramolecular co-pigmentation reactions with different compounds present in the matrix such as some of the phenolic compounds quantified in the products (phenolic acids, catechin, flavonols, etc.) [78]. Overall, the results of this study suggest that not all anthocyanins are equally sensitive to thermal treatment. In Sk + S and S products, peonidin and malvidin derivatives exhibited greater thermal sensitivity than other anthocyanidins. This may be related to their chemical structure, as peonidin and malvidin have only one hydroxyl group in the B ring, whereas the other anthocyanidins have two. Therefore, the latter may be more susceptible to oxidation at high temperatures [79]. Arabinose anthocyanins were the most thermolabile, whereas glucoside anthocyanins were more resistant. However, no clear effect of heat was observed on the aglucone or glycosyl derivatives specific to the S products. These results are consistent with those reported by [80], who investigated the stability of anthocyanins in a bilberry methanolic extract when heated to 80 °C, 100 °C, and 125 °C. The authors observed that arabinose anthocyanins tended to be more thermolabile than their corresponding glucosides or galactosides. Finally, it should be remark that anthocyanin behaviour could be different in the products were used and ingredients in the elaboration of functional foods because of the interactions with some nutrients present in the food (proteins, polysaccharides) which may stabilize these pigments [78].

Considering the results of the microbiological studies, and the previous discussion, only the effect of the thermal treatment at 90 °C and 90 min in the individual phenolic compounds is going to be discussed.

Regarding the effect of the thermal treatment on individual phenolic compounds, it was observed that thermal treatment increased the concentration of all phenolic acids and their derivatives. The increase was much higher in S products than in Sk and Sk + S products, highlighting the significant increase in phenolic acids, particularly benzoic acids (gallic acid, protocatechuic acid, o-methyl gallate and syringic acid), as well as chlorogenic acid (Table 5). Del Pino-García et al. [26] also observed a significant increase in total phenolic acid content after applying a similar thermal treatment to a seasoning obtained from red grape pomace. They found that the increase was more pronounced in seasonings obtained from red wine pomace seeds than in those obtained from skins or skins and seeds together. This increase in phenolic acids could be due to the release of phenolic compounds from the cell wall due to the heating treatment. Xu and Chang [81] established that the application of heat treatment to plants can modify the lignocellulosic and protein structure of the cell wall through dehydration and the initiation of chemical reactions, leading to the disruption of plant tissue and the release of different phenolic compounds into the matrix. Furthermore, these compounds can also be released from the food matrix due to heat [75,76,82]. It is well-known that polyphenols can bind to various compounds in the food matrix, including proteins and polysaccharides. The mechanisms responsible for the formation of protein–polyphenol and polysaccharide–polyphenol complexes are quite similar and include both non-covalent and covalent bonds [83,84]. The former is weaker and can be reversible, resulting in the release of bound polyphenols. In this sense, Chamorro et al. [75] reported that autoclaving grape pomace and grape seed extracts resulted in a shift from highly polymerised to relatively less-polymerised molecules due to the degradation of polymerised procyanidins. Furthermore, heat can lead to the depolymerisation of phenolic compounds. The increase in gallic acid observed in this study may be due to the release of this acid resulting from the excision of the gallate group attached to the C-ring of flavonoids or from the hydrolysis of gallotannins present in the product [75]. Some statistically significant differences were found regarding flavonols, but the differences between flavonols and products were variable and sometimes moved in opposite directions. This made it difficult to establish a general effect of heating. Opposite results have been published previously. So, while Del Pino-García et al. [26] found a significant increase in flavonol content after thermal treatment of a powdered product derived from red grape pomace, Howard et al. [85] observed a decrease in quercetin derivatives after thermal treatment of blueberry pulp from *Vaccinium meridionale*. For the two flavanols studied, the results varied between products and in opposite directions. The observed increases in the Sk and S products for the proanthocyanidin B and in the SK + S and S products for epicathechin may be due to the hydrolysis of tannins as a consequence of thermal treatments, that is, larger oligomers are converted into mono and dimers in response to the thermal treatment [76,86]. Alternatively, they may be released from the former compounds through non-covalent interactions with polysaccharides, particularly pectin [77] as has been explained before. It should also be considered that high-molecular-weight procyanidins may bind irreversibly to cell wall polysaccharides through covalent bonds (i.e., hydrogen bonding and/or hydrophobic interactions), meaning they cannot be released or converted into smaller compounds [83]. On the contrary, the observed decrease in proanthocyanidins in the SK+ S products could be attributed to a possible thermal degradation, in agreement with the results of Del Pino-García et al. [26], who found that heating negatively affected the total flavan-3-ol content. These degradation of proantocyanidins may be due to either insufficient enzyme inactivation resulting in oxidation reactions, or the fact that the released monomers are more prone to non-enzymatic reactions after heating [87].

## 4. Conclusions

The waste generated during the production of blueberry juice can be processed using an environmentally friendly method to produce functional ingredients rich in fibre and phenolic compounds, which can be used in the production of healthier foods. However, to ensure the food safety of these products, it is necessary to carry out a pre-treatment that reduces their initial microbiological load. A heat treatment at 90 °C for 90 min is sufficient to achieve this objective in residues obtained from the whole pomace or just the skins, without significantly affecting their phenolic composition and antioxidant activity. The results obtained in this study enable to tackle new challenges, such as evaluating the products technological properties (water-holding capacity, etc.), their effect on the sensorial properties of the products (colour, texture, taste…) and their final acceptation by consumers, as well as their preservative effect, both to prevent or delay lipid oxidation and to inhibit the growth of spoilage microorganisms, improving food safety by inhibiting foodborne pathogens. Furthermore, new studies about the bioavailability and bioactivity of polyphenols could help to expand the understanding of bioactive compounds in the field of health.

## Figures and Tables

**Table 1:** Proximate composition of blueberry pomace products. (Average ± standard deviation).

Parameter	Sk + S	Sk	S
Humidity (%)	4.02 ± 0.72 ^a^	2.50 ± 0.65 ^a^	2.71 ± 0.10 ^a^
pH	3.52 ± 0.03 ^a^	3.50 ± 0.01 ^a^	3.98 ± 0.00 ^b^
Acidity (g citric acid/100 g)	1.92 ± 0.01 ^a^	1.73 ± 0.27 ^a^	1.41 ± 0.01 ^a^
Proteins (%) DM	10.80 ± 0.60 ^a^	11.00 ± 0.10 ^a^	13.60 ± 0.20 ^b^
Fat (%) DM	14.20 ± 0.10 ^a^	13.70 ± 0.10 ^a^	21.00 ± 0.40 ^b^
Reducing sugars (g/100 g)	8.63 ± 0.53 ^b^	7.94 ± 0.31 ^b^	4.25 ± 0.71 ^a^
Total dietary fibre (%) DM	68.20 ± 1.00 ^b^	69.30 ± 0.30 ^b^	64.80 ± 0.50 ^a^

Different letters denote significant differences (LSD test and *p* < 0.05) between products. Sk + S: product obtained from the whole pomace (skins and seeds); Sk: product obtained from skins; S: product obtained from seeds. DM: dry matter.

**Table 2:** Mineral content of blueberry pomace products. The results are expressed as mg/kg of powder product (ppm). (Average ± standard deviation).

Product	P	Ca	K	Mg	Fe	Mn	Zn	Cu	Na
Sk + S	2166 ± 28 ^a^	1668 ± 8 ^a^	1525 ± 57 ^a^	618 ± 4 ^a^	118 ± 6 ^b^	100 ± 4 ^a^	14.1 ± 1.3 ^a^	12.2 ±0.7 ^a^	4.65 ± 0.07 ^b^
Sk	2112 ± 23 ^a^	1671 ± 30 ^a^	1420 ± 13 ^a^	586 ± 10 ^a^	131 ± 1 ^c^	103 ± 1 ^a^	15.4 ±0.4 ^a^	11.9 ± 0.1 ^a^	3.91 ± 0.29 ^a^
S	2895 ±81 ^b^	1741 ±62 ^b^	1659 ± 77 ^a^	1040 ± 31 ^b^	100 ± 2 ^a^	139 ± 3 ^b^	20.6 ± 0.7 ^b^	12.2 ± 1.0 ^a^	11.70 ± 0.2 ^c^

Different letters denote significant differences (LSD test and *p* < 0.05) between products. Sk + S: product obtained from the whole pomace (skins and seeds); Sk: product obtained from skins; S: product obtained from seeds. DM: dry matter.

**Table 3:** Peak identification of anthocyanins in blueberry samples using HPLC-ESI-QTOF in the three products studied.

Compound	Theoretical Mass (*m*/*z*)	[M]^+^	Error (ppm)	Fragment Ion (*m*/*z*)	Identification Method
Dp-3-galactoside	465.1033	465.1034	2.15	303.0502	UV-V/MS-MS/lit/st
Dp-3-glucoside	465.1033	465.1032	0.25	303.0503	UV-V/MS-MS/lit/st
Cy-3-galactoside	449.1084	449.1075	−2.00	287.0541	UV-V/MS-MS/lit/st
Dp-3-arabionoside	435.0927	435.0929	4.60	303.0500	UV-V/MS-MS/lit
Cy-3-glucoside	449.1084	449.1070	−3.12	287.0547	UV-V/MS-MS/lit/st
Pt-3-galactoside	479.1190	479.1175	−3.13	317.0655	UV-V/MS-MS/lit
Cy-3-arabionoside	419.0978	419.0979	0.24	287.0547	UV-V/MS-MS/lit/st
Pt-3-glucoside	479.1190	479.1175	−3.13	317.0671	UV-V/MS-MS/lit/st
Pn-3-galactoside	463.1240	463.1241	0.22	301.0724	UV-V/MS-MS/lit
Pt-3-arabinoside	449.1078	449.1080	0.45	317.0659	UV-V/MS-MS/lit
Pn-3-glucoside	463.1240	463.1241	0.02	301.0705	UV-V/MS-MS/lit/st
Mv-3-galactoside	493.1346	493.1331	−3.04	331.0812	UV-V/MS-MS/lit
Pn-3-arabinoside	433.1135	433.1133	−0.05	301.0703	UV-V/MS-MS/lit
Mv-3-glucoside	493.1346	493.1331	−3.04	331.0812	UV-V/MS-MS/lit/st
Mv-3-arabionóside	463.124	463.1223	−3.67	331.0813	UV-V/MS-MS/lit
Dp-3-(6″coumarylglucoside)	611.1401	611.1394	−1.14	303.0492	UV-V/MS-MS/lit
Cy-3-(6″coumarylglucoside)	595.1452	595.1438	−2.35	287.0538	UV-V/MS-MS/lit
Mv-3-(6″coumarylglucoside)	639.1714	639.1689	−3.91	331.0797	UV-V/MS-MS/lit

UV-V: ultraviolet-visible. Lit: literature; st: standard.

**Table 4:** Anthocyanin content in blueberry pomace powdered products (Sk + S, Sk and S) without and with thermal treatment at 90 °C for 30, 60, 90 and 120 min. (Average ± standard deviation). The results are expressed as mg/g of sample.

	Delphinidin Derivatives			Cyanidin Derivatives			Petunidin-Derivatives			Peonidin Derivatives			Malvidin Derivatives			Total Anthocyanins
	Gal	Glc	Ara	Gal	Glc	Ara	Gal	Glc	Ara	Gal	Glc	Ara	Gal	Glc	Ara	
Sk + S NT	7.04 ± 0.40 ^b,2^	13.1 ± 1.0 ^b;1,2^	7.69 ± 0.76 ^b,2^	3.97 ± 0.31 ^b,2^	5.85 ± 0.47 ^b,2^	7.09 ± 0.66 ^b,2^	4.79 ± 0.42 ^b,2^	13.0 ±1.2 ^b,2^	3.16 ± 0.24 ^b,2^	0.466 ± 0.026 ^b,3^	2.99 ± 0.22 ^b,2^	0.302 ± 0.014 ^c,3^	1.74 ± 0.11 ^b,2^	5.62 ± 0.42 ^b,2^	0.979 ± 0.069 ^b,3^	77.8 ± 6.1 ^b,2^
Sk + S T30	6.72 ± 0.54 ^1,2^	12.6 ± 1.0 ^1,2^	7.17 ± 0.55 ^2^	3.60 ± 0.25 ^1,2^	5.60 ± 0.39 ^1,2^	6.62 ± 0.47 ^2^	4.62 ± 0.34 ^2^	12.3 ± 0.9 ^1,2^	2.87 ± 0.20 ^2^	0.411 ± 0.032 ^2^	2.81 ± 0.19 ^2^	0.299 ± 0.020 ^3^	1.70 ± 0.13 ^2^	5.32 ± 0.35 ^2^	0.914 ± 0.061 ^3,2^	73.5 ± 5.3 ^2^
Sk + S T60	6.80 ± 0.23 ^1,2^	12.8 ± 0.5 ^1,2^	7.04 ± 0.26 ^1,2^	3.51 ± 0.12 ^1,2^	5.44 ± 0.19 ^1,2^	6.21 ± 0.23 ^2^	4.44 ± 0.15 ^1,2^	12.2 ± 0.4 ^1,2^	2.87 ± 0.12 ^2^	0.362 ± 0.010 ^1,2^	2.73 ± 0.09 ^1,2^	0.309 ± 0.011 ^3^	1.58 ± 0.05 ^2^	5.12 ± 0.18 ^1,2^	0.854 ± 0.034 ^2^	72.2 ± 2.5 ^1,2^
Sk + S T90	7.31 ± 1.04 ^2^	14.0 ± 2.0 ^2^	7.48 ± 1.07 ^2^	3.75 ± 0.56 ^2^	5.89 ± 0.84 ^2^	6.47 ± 0.77 ^2^	4.53 ± 0.48 ^2^	13.2 ± 1.7 ^2^	2.95 ± 0.24 ^2^	0.382 ± 0.046 ^2^	2.98 ± 0.39 ^2^	0.253 ± 0.022 ^2^	1.65 ± 0.20 ^2^	5.48 ± 0.75 ^2^	0.829 ± 0.069 ^2^	77.1 ± 10.2 ^2^
Sk + S T120	5.77 ± 0.55 ^1^	11.3 ± 1.1 ^1^	5.81 ± 0.57 ^1^	3.01 ± 0.03 ^1^	4.76 ± 0.46 ^1^	4.94 ± 0.46 ^1^	3.83 ± 0.36 ^1^	10.7 ± 1.0 ^1^	2.31 ± 0.21 ^1^	0.317 ± 0.011 ^1^	2.34 ± 0.19 ^1^	0.200 ± 0.016 ^1^	1.22 ± 0.10 ^1^	4.32 ± 0.41^1^	0.671 ± 0.055 ^1^	61.5 ± 5.8 ^1^
Sk NT	6.64 ± 0.55 ^b,1^	12.3 ± 1.1 ^b,1^	7.02 ± 0.70 ^b,1^	3.75 ± 0.33 ^b,2^	5.54 ± 0.50 ^b,1^	6.43 ± 0.62 ^b,2^	4.71 ± 0.40 ^b,2^	12.0 ± 1.0 ^b,1^	2.99 ± 0.27 ^b,2^	0.445 ± 0.038 ^b,2^	2.75 ± 0.27 ^b,1^	0.245 ± 0.023 ^b,2^	1.61 ± 0.13 ^b,2^	5.15 ± 0.42 ^b,1^	0.91 ± 0.08 ^b,2^	72.4± 6.5 ^b,2^
Sk T 30	6.16 ± 0.44 ^1^	11.6 ± 0.8 ^1^	6.47 ± 0.45 ^1^	3.25 ± 0.24 ^1^	5.01 ± 0.36 ^1^	5.81 ± 0.42 ^1,2^	4.12 ±0.32 ^1^	11.1 ± 0.8 ^1^	2.57 ± 0.17 ^1,2^	0.355 ± 0.023 ^1^	0.429 ±0.178 ^1^	0.251 ± 0.020 ^2^	1.46 ± 0.10 ^1,2^	4.07 ± 0.34 ^1^	0.808 ± 0.057 ^1,2^	61.9 ± 5.0 ^1^
Sk T 60	6.34 ± 0.51 ^1^	11.9 ± 0.9 ^1^	6.55 ± 0.45 ^1^	3.29 ± 0.24 ^1^	5.07 ± 0.34 ^1^	5.77 ± 0.38 ^1.2^	4.13 ± 0.29 ^1^	11.3 ± 0.8 ^1^	2.59 ± 0.24 ^1,2^	0.353 ± 0.018 ^1^	2.56 ± 0.17 ^1^	0.280 ± 0.022 ^2^	1.48 ± 0.09 ^1,2^	4.77 ± 0.31 ^1^	0.792 ± 0.051 ^1^	67.2 ± 4.8 ^1,2^
Sk T 90	5.87 ± 0.55 ^1^	11.4 ± 0.9 ^1^	6.16 ± 0.52 ^1^	3.16 ± 0.24 ^1^	4.98 ± 0.33 ^1^	5.55 ± 0.42 ^1^	3.90 ± 0.29 ^1^	11.1 ± 0.8 ^1^	2.69 ± 0.33 ^1,2^	0.324 ± 0.027 ^1^	2.58 ± 0.17 ^1^	0.279 ± 0.018 ^2^	1.44 ± 0.10 ^1,2^	4.71 ± 0.30 ^1^	0.766 ± 0.051 ^1^	64.9 ± 5.0 ^1,2^
Sk T-120	5.94 ± 0.31 ^1^	11.5 ± 0.6 ^1^	6.22 ± 0.23 ^1^	3.23 ± 0.10 ^1^	4.95 ± 0.21 ^1^	5.41 ± 0.23 ^1^	4.40 ± 0.15 ^1^	11.0 ± 0.5 ^1^	2.49 ± 0.10 ^1^	0.342 ± 0.010 ^1^	2.46 ± 0.12 ^1^	0.185 ± 0.016 ^1^	1.32 ± 0.06 ^1^	4.57 ± 0.25 ^1^	0.716 ± 0.045 ^1^	64.3 ± 2.9 ^1,2^
S NT	2.47 ± 0.25 ^a,1^	5.59 ± 0.44 ^a,1^	2.80 ± 0.26 ^a;1,2^	1.59 ± 0.14 ^a,2^	2.36 ± 0.20 ^a,2^	2.99 ± 0.26 ^a,3^	1.90 ± 0.17 ^a;1,2^	4.84 ± 0.42 ^a,1^	1.28 ± 0.16 ^a,3^	0.206 ± 0.018 ^a,4^	1.25 ± 0.11 ^a,2^	0.109 ± 0.014 ^a;1,2^	0.735 ± 0.063 ^a,2^	2.3 ± 0.19 ^a;1,2^	0.444 ± 0.037 ^a,3^	29.9 ± 2.7 ^a;1,2^
S T30	2.65 ± 0.16 ^1^	4.94 ± 0.25 ^1^	2.96 ± 0.15 ^2^	1.62 ± 0.08 ^2^	2.45 ± 0.09 ^1,2^	3.03 ± 0.12 ^3^	1.94 ± 0.09 ^2^	5.17 ± 0.21 ^1^	1.28 ± 0.05 ^2,3^	0.192 ± 0.006 ^3,4^	1.25 ± 0.03 ^2^	0.174 ± 0.004 ^2,3^	0.761 ± 0.041 ^2^	2.45 ± 0.08 ^3^	0.443 ± 0.014 ^3^	31.3 ± 1.4 ^2^
S T60	2.54 ± 0.12 ^1^	4.71 ± 0.23 ^1^	2.74 ± 0.14 ^1,2^	1.51 ± 0.07 ^1,2^	2.28 ± 0.12 ^1,2^	2.74 ± 0.15 ^2,3^	1.81 ± 0.10 ^1,2^	4.82 ± 0.25 ^1^	1.12 ± 0.06 ^1,2^	0.174 ± 0.012 ^2,3^	1.21 ± 0.06 ^2^	0.124 ± 0.007 ^3^	0.707 ± 0.038 ^2^	2.24 ± 0.11 ^1,2,3^	0.391 ± 0.019 ^2^	29.1 ± 3.0 ^1,2^
S T90	2.53 ± 0.29 ^1^	4.73 ± 0.51 ^1^	2.68 ± 0.28 ^1,2^	1.48 ± 0.15 ^1,2^	2.24 ± 0.23 ^1,2^	2.61 ± 0.26 ^1,2^	1.78 ± 0.19 ^1,2^	4.79 ± 0.49 ^1^	1.12 ± 0.12 ^1,2^	0.163 ± 0.019 ^1,2^	1.18 ± 0.12 ^1,2^	0.127 ± 0.013 ^4^	0.674 ± 0.066 ^2^	2.18 ± 0.21 ^1,2^	0.363 ± 0.034 ^2^	28.6 ± 3.0 ^1,2^
S 120	2.48 ± 0.18 ^1^	4.61 ± 0.31 ^1^	2.46 ± 0.16 ^1^	1.38 ± 0.07 ^1^	2.10 ± 0.10 ^1^	2.31 ± 0.12 ^1^	1.67 ± 0.10 ^1^	4.53 ± 0.25 ^1^	1.02 ± 0.06 ^1^	0.145 ± 0.008 ^1^	1.06 ± 0.05 ^1^	0.098 ± 0.005 ^1^	0.568 ± 0.029 ^1^	2.00 ± 0.10 ^1^	0.308 ± 0.015 ^1^	26.7 ± 1.6 ^1^

Gal:galactoside; Glu:glucoside; Ara:arabinoside. Different letters denote significant differences (LSD test and *p* < 0.05) between NT products. Different numbers denote significant differences, (LSD test and *p* < 0.05) between thermal treatments. Sk + S: product obtained from the whole pomace (skins and seeds); Sk: product obtained from skins; S: product obtained from seeds. NT: no thermal treatment.

**Table 5:** Polyphenolic content in pomace powdered products (Sk + S, Sk and S) without thermal treatment (NT) and subjected to a thermal treatment of 90 °C for 90 min (TT) (Average ± standard deviation). Results are expressed as mg of quercetin-3-glucoside/g of sample.

	SK + S		SK		S	
Compounds	NT	TT	NT	TT	NT	TT
*Phenolic acids*						
Gallic acid	0.179 ± 0.010 ^b,1^	0.276 ± 0.027 ^b,2^	0.213 ± 0.004 ^b,1^	0.317 ± 0.033 ^c,2^	0.031 ± 0.002 ^a,1^	0.119 ± 0.011 ^a;b,2^
Protocatequic acid.	0.183 ± 0.009 ^b,1^	0.292 ± 0.013 ^b,2^	0.194 ± 0.011 ^b,1^	0.324 ± 0.018 ^c,2^	0.062 ± 0.004 ^a,1^	0.170 ± 0.013 ^a,2^
o-Methyl galate	0.052 ± 0.004 ^b,1^	0.094 ± 0.012 ^b,2^	0.048 ± 0.004 ^b,1^	0.105 ± 0.009 ^b,2^	0.014 ± 0.001 ^a,1^	0.047 ± 0.002 ^a,2^
p-Coumaric acid hexoside	0.012 ± 0.000 ^a,1^	0.017 ± 0.001 ^a,2^	0.014 ± 0.000 ^a,1^	0.016 ± 0.001 ^a,2^	0.016 ± 0.001 ^b,1^	0.019 ± 0.0004 ^b,2^
Caffeic acid	0.052 ± 0.002 ^b,1^	0.061 ± 0.004 ^a,2^	0.049 ± 0.004 ^b,1^	0.065 ± 0.005 ^a,2^	0.042 ± 0.003 ^a,1^	0.055 ± 0.003 ^a,2^
Chlorogenic acid	0.434 ± 0.013 ^b,1^	0.508 ± 0.042 ^b,2^	0.475 ± 0.026 ^c,1^	0.606 ± 0.027 ^c,2^	0.172 ± 0.015 ^a,1^	0.330 ± 0.021 ^a,2^
Syringic acid	0.460 ± 0.044 ^c,1^	0.594 ± 0.045 ^d,2^	0.440 ± 0.011 ^c,1^	0.682 ± 0.083 ^d,2^	0.097 ± 0.002 ^a,1^	0.238 ± 0.016 ^b,2^
p-coumaric acid	0.104 ± 0.005 ^a,1^	0.141 ± 0.007 ^a,2^	0.104 ± 0.007 ^a,1^	0.148 ± 0.007 ^a,1^	0.107 ± 0.005 ^a,1^	0.147 ± 0.008 ^a,2^
Sinapic acid	0.504 ± 0.045 ^b,1^	0.542 ± 0.026 ^a,1^	0.491 ± 0.022 ^b,1^	0.571 ± 0.047 ^a,1^	0.366 ± 0.022 ^a,1^	0.478 ± 0.051 ^a,2^
Total	1.99 ± 0.04	2.52 ± 0.15	2.03 ± 0.06	2.83 ± 0.20	0.905 ± 0.048	1.603 ± 0.121
*Flavonols*						
Myricetin-hexoside 1	0.395 ± 0.033 ^b,1^	0.407 ± 0.047 ^b,1^	0.414 ± 0.025 ^b,1^	0.399 ± 0.024 ^b,1^	0.150 ± 0.008 ^a,1^	0.165 ± 0.018 ^a,1^
Myricetin-hexoside 2	0.112 ± 0.003 ^b,1^	0.120 ± 0.014 ^b,1^	0.115 ± 0.006 ^b,1^	0.110 ± 0.002 ^b,1^	0.038 ± 0.002 ^a,1^	0.046 ± 0.005 ^a,1^
Quercetin-hexoside 1	0.554 ± 0.046 ^c,1^	0.726 ± 0.039 ^b,2^	0.446 ± 0.040 ^b,1^	0.683 ± 0.059 ^b,2^	0.199 ± 0.011 ^a,1^	0.307 ± 0.017 ^a,2^
Quercetin-hexoside 2	0.703 ± 0.038 ^c,2^	0.441 ± 0.041 ^b,1^	0.541 ± 0.041 ^b,2^	0.436 ± 0.037 ^b,1^	0.314 ± 0.017 ^a,2^	0.220 ± 0.0149 ^b,1^
Laricitrin hexoside	0.114 ± 0.014 ^b,1^	0.081 ± 0.018 ^a,1^	0.151 ± 0.017 ^c,1^	0.124 ± 0.009 ^b,1^	0.053 ± 0.007 ^a,1^	0.066 ± 0.002 ^a,2^
Quercetin pentoside	0.072 ± 0.004 ^a,1^	0.093 ± 0.008 ^c,2^	0.071 ± 0.005 ^a,1^	0.079 ± 0.003 ^b,1^	nd	0.046 ± 0.005 ^a^
Myricetin	0.057 ± 0.006 ^b,1^	0.060 ± 0.003 ^b,c;1^	0.059 ± 0.006 ^b,c;1^	0.067 ± 0.003 ^c,1^	nd	0.035 ± 0.002 ^a^
Total	2.01 ± 0.03	2.05 ± 0.26	1.80 ± 0.082	1.90 ± 0.06	0.755 ± 0.035	0.886 ± 0.040
*Flavanols*						
Proanthocnidin B2	0.400 ± 0.036 ^b,1^	0.188 ± 0.018 ^a,2^	0.509 ± 0.044 ^b,1^	0.633 ± 0.086 ^c,2^	0.195 ± 0.027 ^a,1^	0.242 ± 0.031 ^a,1^
Epicatequin	0.046 ± 0.003 ^a,1^	0.095 ± 0.014 ^a,2^	0.123 ± 0.005 ^b,1^	0.116 ± 0.009 ^a,1^	0.066 ± 0.007 ^a,1^	0.096 ± 0.007 ^a,2^
Total	0.446 ± 0.050 ^2^	0.283 ± 0.017	0.632 ± 0.047	0.749 ± 0.080	0.261 ± 0.026	0.339 ± 0.038

Different letters denote significant differences (LSD test and *p* < 0.05) between the six products without taken the thermal treatment into account. Different numbers denote significant differences (LSD test and *p* < 0.05) between thermal treatments for each product. Sk + S: product obtained from the whole pomace (skins and seeds); Sk: product obtained from skins; S: product obtained from seeds; nd: not detected. NT: no thermal treatment.

**Table 6:** Peak identification of phenolic compounds in blueberry samples using HPLC-ESI-QTOF in the three products studied.

Compound	DAD λ_max_ (nm)	Theoretical Mass (*m*/*z*)	[M-H]^−^	Error Ppm	Fragment Ion (*m*/*z*)	Identification
Gallic acid	280	170.1215	169.0139	−0.9000	125.0239	UV-V/MSMS/st
Protocatequic acid	254	154.0268	153.0196	−1.0900	109.2870	UV-V/MSMS/st
o-Methyl gallate	280	184.0372	183.0294	2.7000	124.0165	UV-V/MSMS
p-coumaric acid hexoside	320	326.1002	325.0921	2.7000	163.0405	UV-V/MSMS
Caffeic acid	320	180.0423	179.0343	3.7900	135.0453	UV-V/MSMS/st
Myricetin-hexoside 1	360	480.0904	479.0845	2.5500	317.0280	UV-V/MSMS
Myricetin hexoside 2	360	480.0904	479.0846	−3.0900	317.0324	UV-V/MSMS
Chlorogenic acid	320	354.0951	353.0885	−1.9600	191.0570	UV-V/MSMS/stt
Syringic acid	280					UV-V/st
Proanthocyanidin B2	280	578.1424	577.1323	4.9300	289.0721	UV-V/MSMS
Epicatechin	280	290.0790	289.0726	−2.8900	245.0830	UV-V/MSMS
p-Coumaric acid	320	164.0473	163.0396	2.8500	137.0242	UV-V/MSMS/st
sinapic acid	320					UV-V/st
Quercetin-hexoside 1	360	464.0955	463.0897	3.2300	300.0283	UV-V/MSMS
Quercetin-hexoside 2	360	464.0955	463.0882	0.0000	300.0276	UV-V/MSMS
Laricitrin hexoside	360	494.1060	493.0980	1.5500	331.0460	UV-V/MSMS
Quercetin-pentoside	360	434.0849	433.0770	1.4600	300.0280	UV-V/MSMS
Myricetin	360	318.0376	317.0300	0.9100	178.9983	UV-V/MSMS

UV-V: ultraviolet-visible; st: standard.

**Table 7:** Microbial characterization of the pomace powdered products (Sk + S, Sk and S) without thermal treatment.

Product	AMB *	LAB *	*Enterobactericeae*	*Bacillus* spp.	*Clostridium* spp.	Moulds and Yeast
Sk + S	1.98 ± 0.15 ^a^ **	2.31 ± 0.17	<1.11 ^++^	2.21 ± 0.18	<1.11	2.75 ± 0.73
Sk	1.76 ± 0.07 ^a^	<2.11 ^+^	<1.11	2.41 ± 0.00	<1.11	2.11 ± 0.00
S	3.37 ± 0.98 ^b^	2.11	3.01 ± 0.08	2.41 ± 0.00	<1.11	<2.11

* AMB: aerobic mesophilic bacteria; LAB: lactic acid bacteria; ** results are expressed as log cfu/g ± standard deviation; ^+^ quantification limit in the streaking plate method; ^++^ quantification limit in the pouring plate method; Different letters denote significant differences (LSD test and *p* < 0.05) between NT products; Sk + S: product obtained from the whole pomace (skins and seeds); Sk: product obtained from skins; S: product obtained from seeds.

## Data Availability

The original contributions presented in this study are included in the article/Supplementary Material. Further inquiries can be directed to the corresponding author.

## Supplementary Materials

The following supporting information can be downloaded at https://www.mdpi.com/article/10.3390/foods15101800/s1, Figure S1: UV-Vis spectra of the quantified anthocyanins, Figure S2: UV-Vis spectra of the quantified phenolic compounds.

## Abbreviations

The following abbreviations are used in this manuscript:

ABTS	2,2′-azinobis-(3-ethylbenzothiazoline-6-sulphonic acid)
AMB	Aerobic Mesophilic Bacteria
DAD	Diode array detector
ESI	Electrospray Ionization
HPLC	High Perfomance Liquid Chromatography
ICP-OES	Optical Emission Spectrometry
LAB	Lactic Acid Bacteria
LSD	Least Significant Difference
Q-TOF	Quadrupole Time of Fly
SkS	Pomace with skins and seeds
Sk	Pomace without seeds
S	Seeds
