# Supplementary material for: Physical–Chemical and Microbiological Characterisation of Blueberry By-Products (Vaccinium myrtillus L.) as Potential Food Ingredients
Source: Foods. 2026 May 19;15(10):1800. doi: 10.3390/foods15101800 (PMC13206334; doi:10.3390/foods15101800)
Supplement: Supplementary file 1 [file foods-15-01800-s001.zip › foods-4289351-supplementary.pdf]

Article

Physical–Chemical and Microbiological Characterisation of Blueberry By-Products (*Vaccinium myrtillus* L.) as Potential Food Ingredients

Miriam Ortega-Heras *, Mª Luisa González-Sanjosé, Ruth Hortigüela-Delgado, Ángela Fernández-Varona, Verónica Rodríguez and Beatriz Melero

Department of Biotechnology and Food Science, University of Burgos, Plaza Misael Bañuelos, 09001 Burgos, Spain; marglez@ubu.es (M.L.G.-S.); bmelero@ubu.es (B.M.)

* Correspondence: miriorte@ubu.es

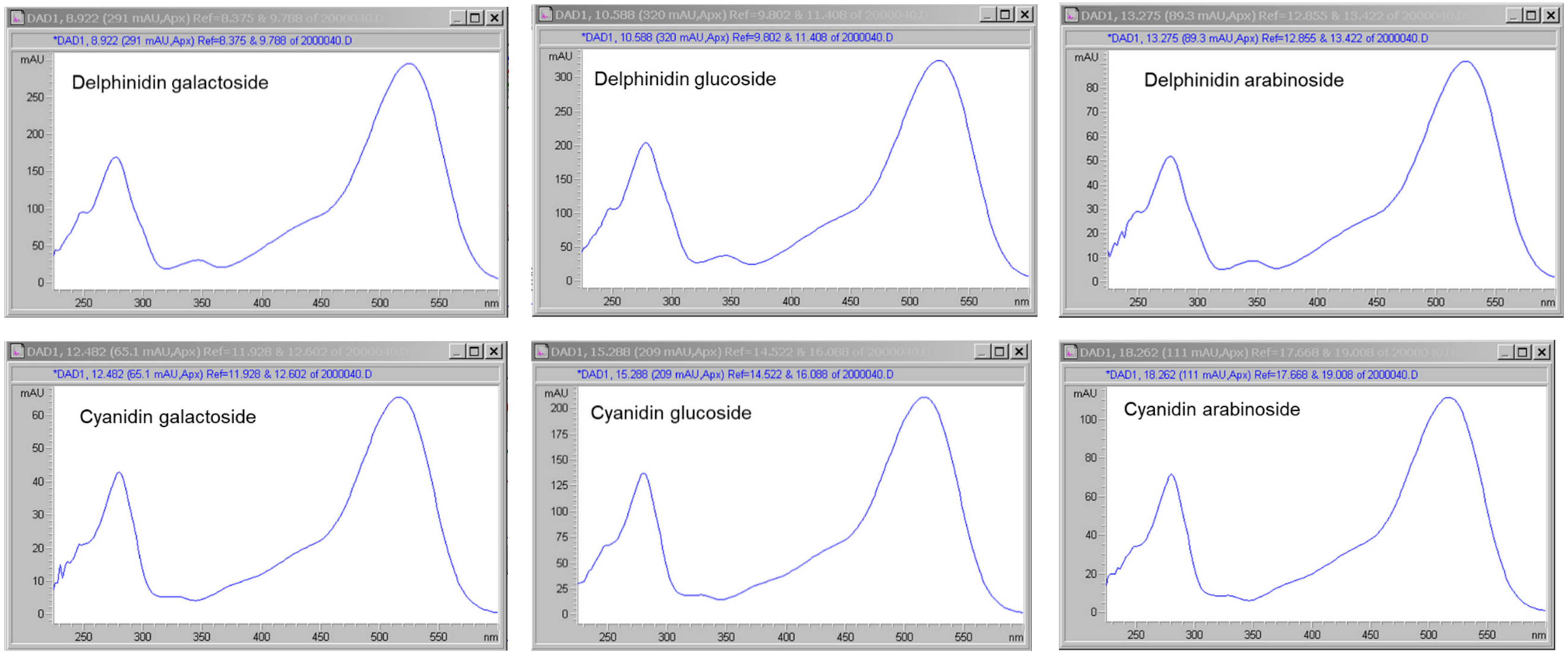

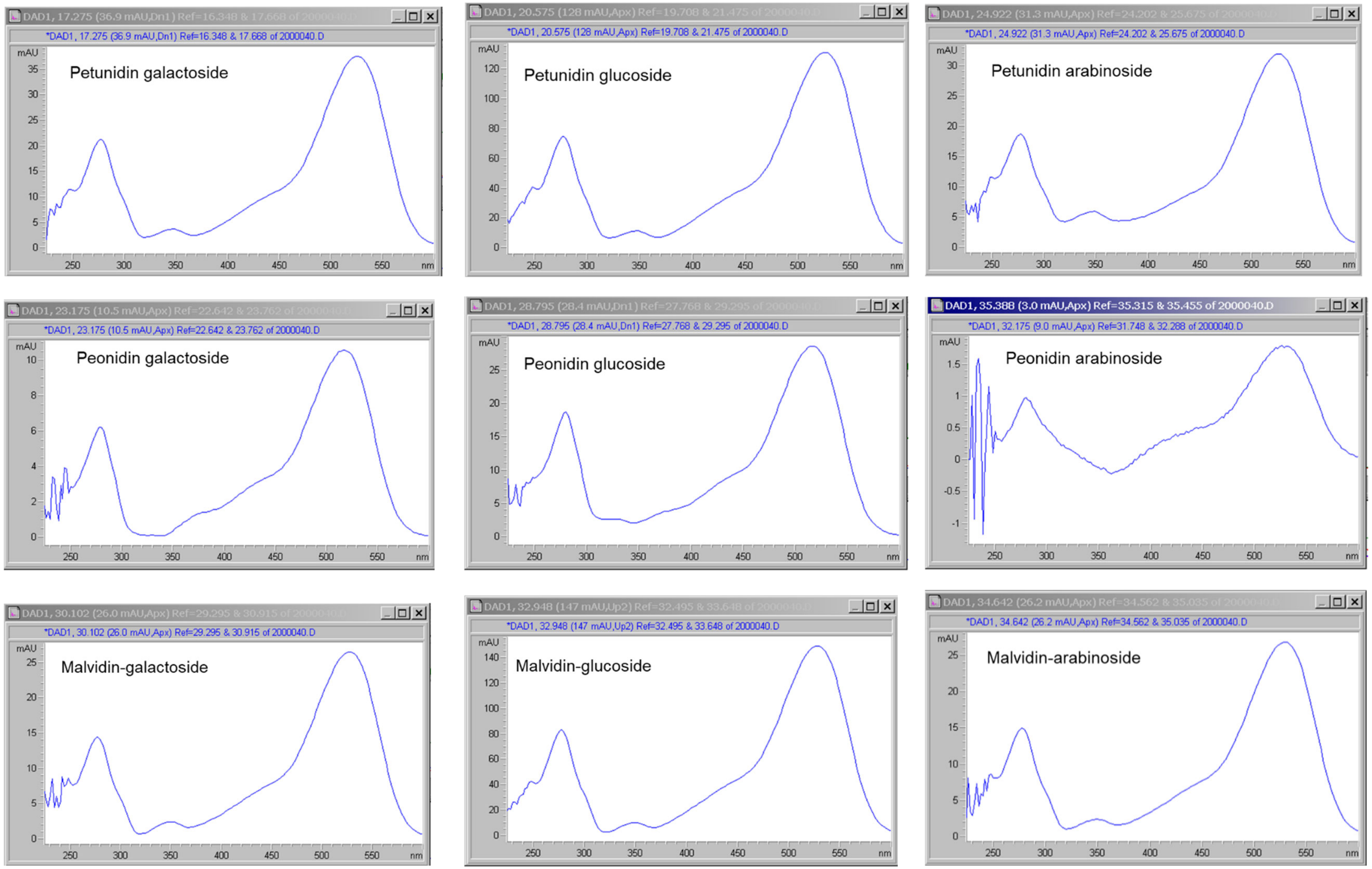

Figure S1: UV-Vis spectra of the quantified anthocyanins.

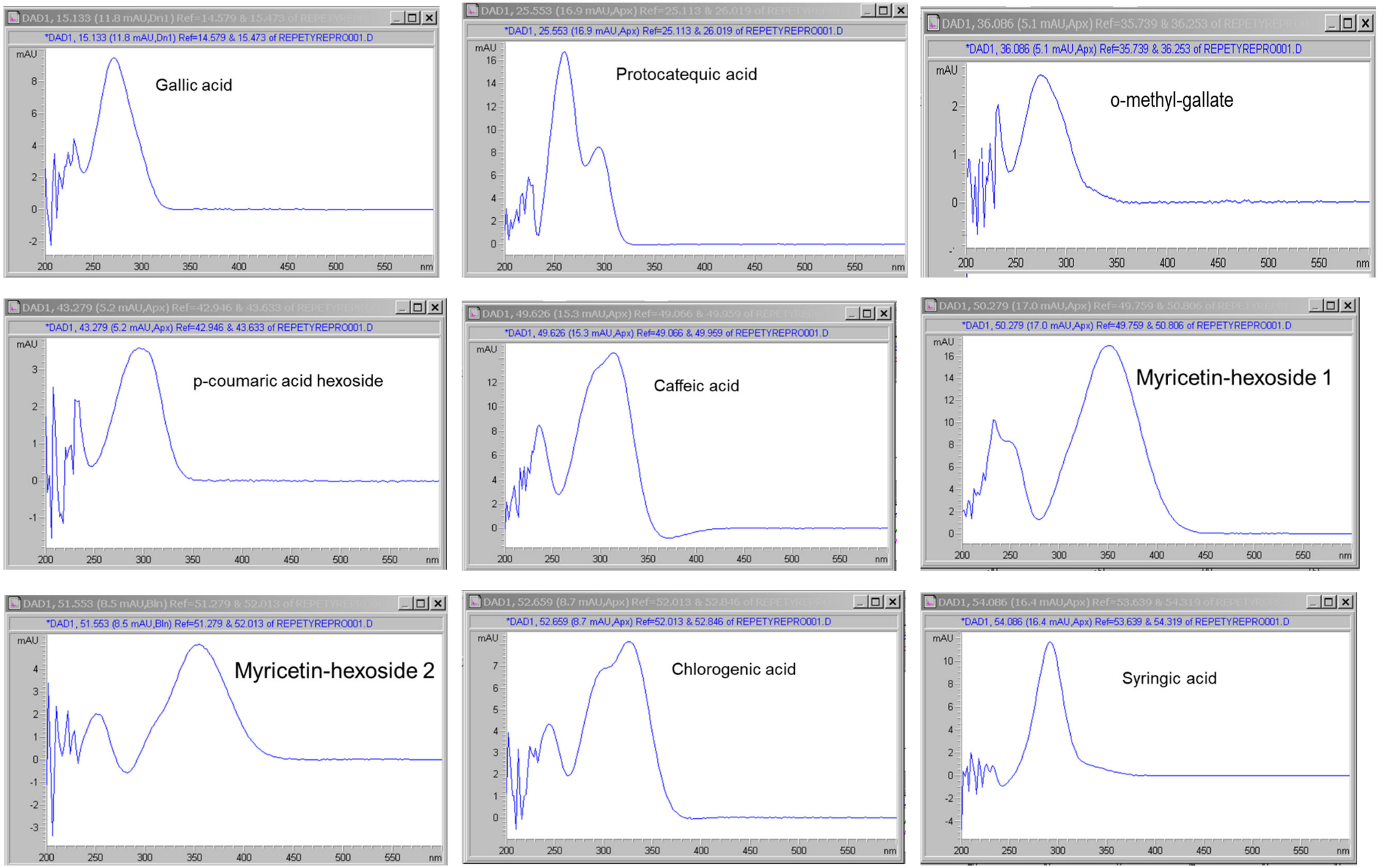
*DAD1, 15.133 (11.8 mAU,Dn1) Ref=14.579 & 15.473 of REPETYREPRO001.D
Gallic acid
*DAD1, 25.553 (16.9 mAU,Apx) Ref=25.113 & 26.019 of REPETYREPRO001.D
Protocatequic acid
*DAD1, 36.086 (5.1 mAU,Apx) Ref=35.739 & 36.253 of REPETYREPRO001.D
o-methyl-gallate
*DAD1, 43.279 (5.2 mAU,Apx) Ref=42.946 & 43.633 of REPETYREPRO001.D
p-coumaric acid hexoside
*DAD1, 49.626 (15.3 mAU,Apx) Ref=49.066 & 49.959 of REPETYREPRO001.D
Caffeic acid
*DAD1, 50.279 (17.0 mAU,Apx) Ref=49.759 & 50.806 of REPETYREPRO001.D
Myricetin-hexoside 1
*DAD1, 51.553 (8.5 mAU,Bln) Ref=51.279 & 52.013 of REPETYREPRO001.D
Myricetin-hexoside 2
*DAD1, 52.659 (8.7 mAU,Apx) Ref=52.013 & 52.846 of REPETYREPRO001.D
Chlorogenic acid
*DAD1, 54.086 (16.4 mAU,Apx) Ref=53.639 & 54.319 of REPETYREPRO001.D
Syringic acid
mAU
nm

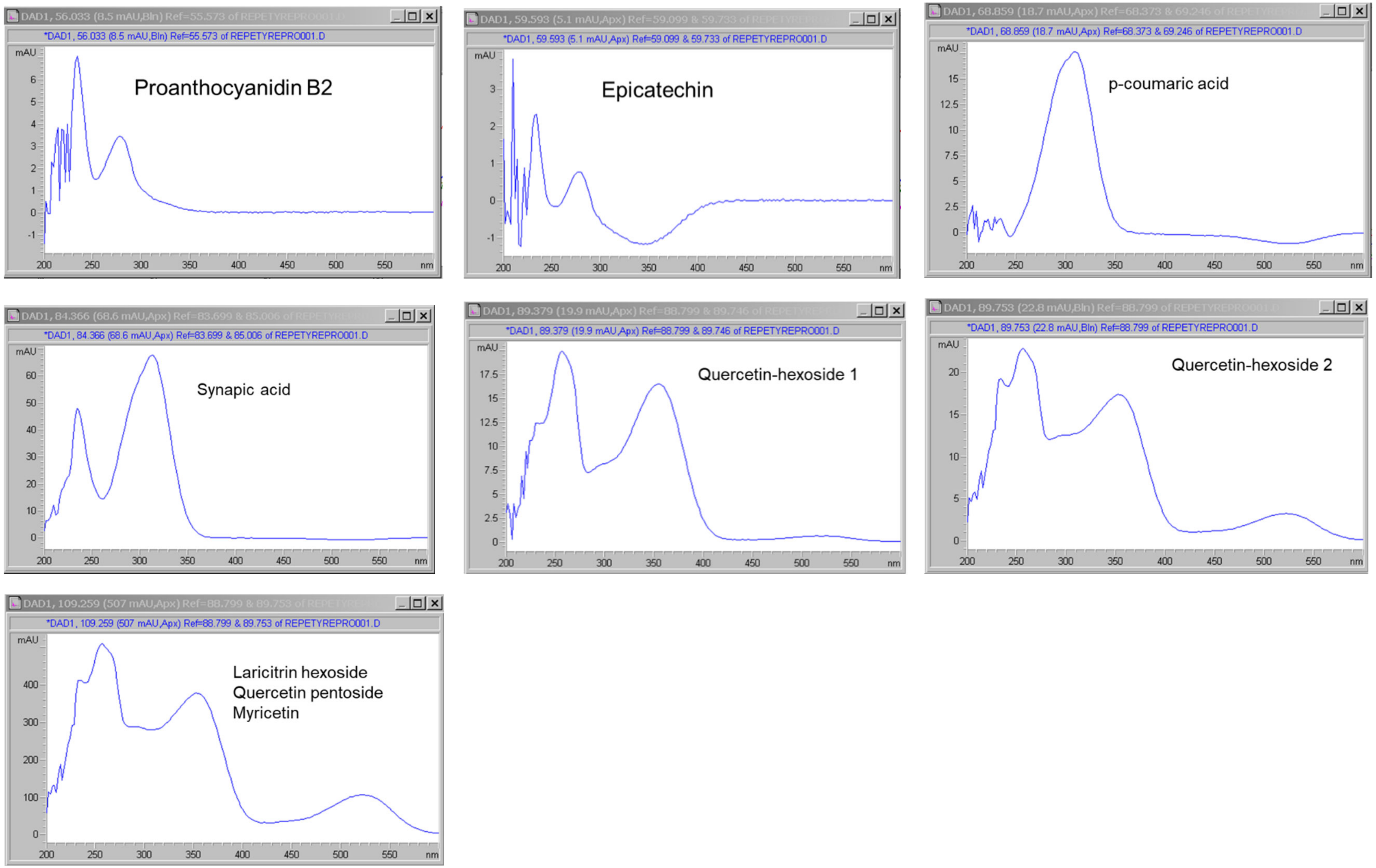

Figure S2: UV-Vis spectra of the quantified phenolic compounds.
